# Obituary: Arnold Hasselblatt (1929 - 2024)

**DOI:** 10.1007/s00210-025-03890-w

**Published:** 2025-02-15

**Authors:** Ingo Rustenbeck, Hans-Georg Joost, Elke Oetjen, Wolfgang Poser

**Affiliations:** 1 Institut für Pharmakologie, Toxikologie und Klinische Pharmazie, Technische Universität Braunschweig, Mendelssohnstraße 1, D-38106 Braunschweig, Germany; 2 Deutsches Institut für Ernährungsforschung Potsdam-Rehbrücke, Arthur-Scheunert-Allee 114-116, D-14558 Nuthetal, Germany; 3 Institut für Klinische Pharmakologie, Pharmakologie für Pharmazeuten, Universitätsklinikum Hamburg-Eppendorf, Martinistraße 52, 20246 Hamburg, Germany; 4 Klinik für Psychiatrie und Psychotherapie der Universitätsmedizin Göttingen, von-Siebold-Straße 5, D-37075 Göttingen, Germany


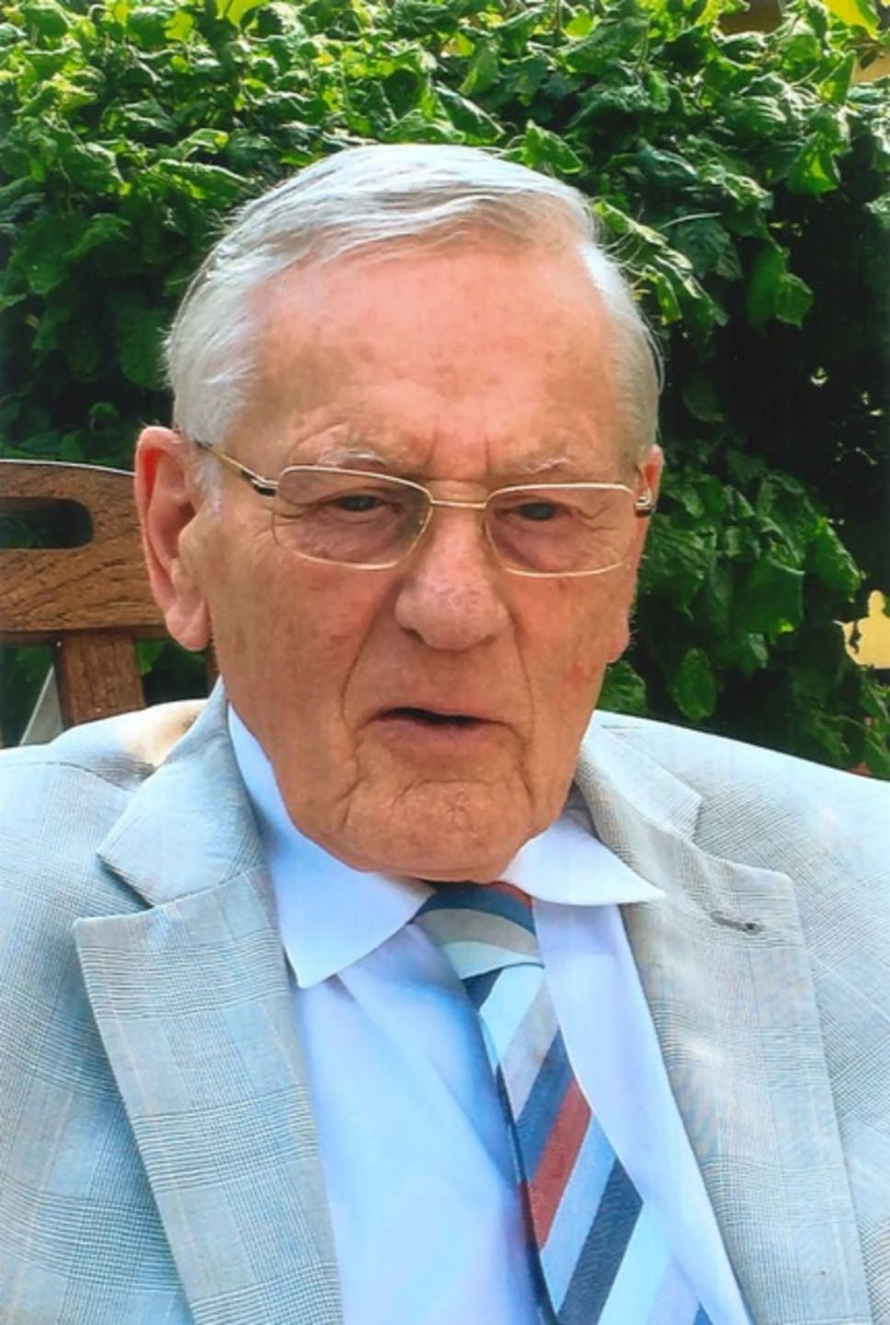


Arnold Hasselblatt was born in Reval (now Tallin) / Estonia in 1929 to a German-speaking family of Swedish ancestry. In 1939 the family was forced to emigrate to Poznan which was at that time occupied by Nazi Germany. At the end of WW II the family fled to West Germany and resettled in Göttingen. Having studied medicine at the Georgia Augusta University of Göttingen, he chose to pursue a scientific career at the Institute of Pharmacology where he had completed his medical thesis work under the supervision of Prof. Ludwig Lendle in 1957. Lendle was an influential figure in the re-structuring of the German science organization in the post-war period who sought to re-establish international contacts. Based on a postdoctoral research grant Arnold Hasselblatt was able to spend one year at the National Medical Research Institute at Mill Hill / London in the department headed by Wilhelm Feldberg. Feldberg was one of the numerous Jewish pharmacologists who were dismissed in 1933 and emigrated from Germany (Mispagel and Seifert 2024). After the war, he fostered the exchange between German and British pharmacologists.

Upon his return to Göttingen Arnold Hasselblatt took up work on the mechanisms of action of the newly described oral antidiabetic drugs, the sulfonylureas. In 1962 the habilitation for pharmacology was awarded for his thesis “Untersuchungen über das Auftreten von gebundenem Insulin im Serum und seine Aktivierung durch Tolbutamid in vitro (investigations on the occurrence of bound insulin in serum and its activation by tolbutamide in vitro)”. Ever since, his interests centered on the pathophysiology and the pharmacological treatment of diabetes mellitus, primarily type 2 diabetes. His early work on the interaction of the carbohydrate and lipid metabolism in the liver was stimulated by the observation that the inhibition of lipolysis by 3,5-dimethylisoxazol or, in short, “Isox” led to a decrease in blood glucose levels (Poser et al. 1969). These experiments, which were performed in collaboration with Ulrich Schwabe and Wolfgang Poser, ultimately led to the concept of an antidiabetic drug with a dual mechanism of action, the stimulation of insulin secretion combined with the inhibition of lipolysis. Theoretically, this aim was achieved by the synthesis of a sulfonylurea with an isoxazol moiety, glisoxepid. While this compound was safe and effective, its blood glucose-lowering efficacy was not superior to that of glibenclamide (glyburide), the commonly used sulfonylurea at that time, so the pharmaceutical producer ultimately lost interest.

In 1967 Uwe Panten joined the group and investigated the metabolism of single perifused pancreatic islets by measuring their NAD(P)H- and FAD-autofluorescence (Panten et al. 1973), a method previously used for the investigation of the hepatic metabolism. These results, combined with the measurement of insulin secretion, tipped the balance of evidence in favor of the substrate site hypothesis which provided that for glucose to stimulate insulin secretion it has to be metabolized. This remarkable advance was made possible by the contact with Bo Hellman´s group at Umea University in Sweden where a colony of ob/ob mice existed. The pancreatic islets of these mice have a tenfold larger volume and consist nearly entirely of beta-cells, which allowed clear-cut interpretations with the microscopic equipment available in the nineteen seventies.

In 1971, Arnold Hasselblatt was appointed full professor of Pharmacology and head of the institute of Pharmacology in Göttingen. This unusual move by the Georgia Augusta medical faculty to promote a group leader to a professorship at  the same institute was made possible by concurrent offers for professorships from other universities (Frankfurt, Tübingen). Still interested in the role of metabolic signals in regulating organ function he asked Sigurd Lenzen to investigate the relation between thyroid function and the endocrine pancreas (Lenzen et al. 1975), since hyperthyroidism was known to interfere with glucose homeostasis.

Together with Hans-Georg Joost, Arnold Hasselblatt investigated the inhibitory effects of tricyclic compounds such as cyproheptadine, amitryptilin or doxepin, on beta cell function (Joost et al. 1976). When the ATP-sensitive K-channel was identified as the link between metabolism and electrical activity of the beta cell, the question came up as to whether imidazoline compounds modulate insulin secretion via alpha adrenoceptors or direct inhibition of the channel or imidazoline-preferring binding sites. This project was entrusted to Ingo Rustenbeck (Rustenbeck et al. 1997).

This overview on Arnold Hasselblatt´s scientific interests shows how he structured the work in the institute. Postdocs were allocated to separate research projects on the endocrine pancreas and, with growing experience, were expected to develop full independence. Intra-institute collaboration of groups was welcome and sometimes very successful such as Uwe Panten´s and Sigurd Lenzen´s collaboration to clarify the metabolic pathways which underlie the insulinotropic effect of certain keto acids and amino acids (Lenzen et al. 1982). The young scientists could publish independently, and it is remarkable that Arnold Hasselblatt often refused to co-author their publications when he felt that his own contribution did not merit it.

From 1982-1983, Arnold Hasselblatt was president of the German Diabetes Society (DDG). From 1983 to 1986, he was president of the German Society of Pharmacology, Toxicology and Clinical Pharmacology (DGPT). Both societies elected him as honorary member. Within the DGPT, he strove to preserve the unity of the society. To foster the interaction of pharmacology with clinical medicine, he lobbied for the establishment of professorships of clinical pharmacology at each medical faculty in Germany. Likewise, the mutual exchange with clinical medicine was the driving force for his engagement in the German Diabetes Society. His view that treatment of multifaceted diseases such as type 2 diabetes often requires combinations of agents with different mechanisms of action is widely accepted. Also, metabolic pathways are now accepted as potential targets for intervention.

In teaching pharmacology, bridging the gap between the experimental approach of the natural sciences and the empirical approach of clinical medicine, was of particular relevance for him. Together with Gerhard Schmidt he advocated the use of multiple choice examinations to overcome the often subjective assessment of the students´ performance in oral examinations. This was not only relevant for the medical faculty in Göttingen: for years, the senior scientists of the institute supported the Institute for Medical Examination Questions (IMPP) by providing questions for the state examinations of medical students in Germany.

When the Soviet Union collapsed and Estonia became an independent state again, Arnold Hasselblatt contacted scientists of his country of birth. This resulted in the cooperation between the University of Göttingen and the University of Tartu (formerly named Dorpat) where the first professorship for Pharmacology in Europe had been established in the nineteenth century (Philippu and Seifert 2023; Toomsalu 2023). Due to his initiative, a number of students from Tartu could come every year and spend a time at the University of Göttingen. In recognition of these efforts, which continued long after his retirement, he was awarded an honorary doctorate by the University of Tartu.

Considering Arnold Hasselblatt´s legacy for his disciples and fellow scientists, it is not so much about specific topics and publications but about attitudes: Firstly, a scientist must be the most critical reviewer of his own data and concepts. Secondly, the conceptual independence of the junior group leader is the mainstay of scientific inquiry. Thirdly, support of junior scientists is a long-term engagement and must not depend on short-term success or failure. These principles guided him throughout his academic career and beyond.

Arnold Hasselblatt passed away on November 24, 2024 at the age of 95. We have lost an outstanding scientist and a committed academic teacher, a warm personality and a brilliant mind. He was orientation and guidance for us, and we are deeply grateful for the mentorship and support we received from him throughout our careers. Our heartfelt condolences go out to his family.
